# Investigating circulating tumor cells and distant metastases in patient-derived orthotopic xenograft models of triple-negative breast cancer

**DOI:** 10.1186/s13058-019-1182-4

**Published:** 2019-08-28

**Authors:** Vishnu C. Ramani, Clementine A. Lemaire, Melanie Triboulet, Kerriann M. Casey, Kyra Heirich, Corinne Renier, José G. Vilches-Moure, Rakhi Gupta, Aryana M. Razmara, Haiyu Zhang, George W. Sledge, Elodie Sollier, Stefanie S. Jeffrey

**Affiliations:** 1 Department of Surgery, Stanford University School of Medicine, Stanford, CA USA; 2 Vortex Biosciences Inc., Pleasanton, CA USA; 3 Department of Comparative Medicine, Stanford University School of Medicine, Stanford, CA USA; 4 Department of Medicine, Stanford University School of Medicine, Stanford, CA USA

**Keywords:** Circulating tumor cells (CTCs), Epithelial-mesenchymal transition (EMT), Liquid biopsy, NOD *scid* gamma (NSG), Patient-derived orthotopic xenograft (PDOX), Triple-negative breast cancer (TNBC)

## Abstract

**Background:**

Circulating tumor cells (CTCs) represent a temporal “snapshot” of a patient’s cancer and changes that occur during disease evolution. There is an extensive literature studying CTCs in breast cancer patients, and particularly in those with metastatic disease. In parallel, there is an increasing use of patient-derived models in preclinical investigations of human cancers. Yet studies are still limited demonstrating CTC shedding and metastasis formation in patient-derived models of breast cancer.

**Methods:**

We used seven patient-derived orthotopic xenograft (PDOX) models generated from triple-negative breast cancer (TNBC) patients to study CTCs and distant metastases. Tumor fragments from PDOX tissue from each of the seven models were implanted into 57 NOD *scid* gamma (NSG) mice, and tumor growth and volume were monitored. Human CTC capture from mouse blood was first optimized on the marker-agnostic Vortex CTC isolation platform, and whole blood was processed from 37 PDOX tumor-bearing mice.

**Results:**

Staining and imaging revealed the presence of CTCs in 32/37 (86%). The total number of CTCs varied between different PDOX tumor models and between individual mice bearing the same PDOX tumors. CTCs were heterogeneous and showed cytokeratin (CK) positive, vimentin (VIM) positive, and mixed CK/VIM phenotypes. Metastases were detected in the lung (20/57, 35%), liver (7/57, 12%), and brain (1/57, less than 2%). The seven different PDOX tumor models displayed varying degrees of metastatic potential, including one TNBC PDOX tumor model that failed to generate any detectable metastases (0/8 mice) despite having CTCs present in the blood of 5/5 tested, suggesting that CTCs from this particular PDOX tumor model may typify metastatic inefficiency.

**Conclusion:**

PDOX tumor models that shed CTCs and develop distant metastases represent an important tool for investigating TNBC.

**Electronic supplementary material:**

The online version of this article (10.1186/s13058-019-1182-4) contains supplementary material, which is available to authorized users.

## Background

Despite the tremendous progress made in the diagnosis and treatment of breast cancer, tumors of the breast still remain one of the leading causes of cancer-related deaths in women [1]. The intertumoral and intratumoral molecular heterogeneity of breast cancer challenges its diagnosis and effective treatment [2–9]. Tailored therapies, such as hormone therapies (e.g., tamoxifen and inhibitors of the enzyme aromatase, involved in estrogen synthesis) for ER-positive disease and trastuzumab (Herceptin®) for HER2-overexpressing breast cancer have led to considerable success in treating some subtypes of breast cancer. However, drug resistance to these regimens can represent a major hurdle to successful treatment [10–15]. Most importantly, there is still no good targeted therapy for triple-negative breast cancer (TNBC), a very aggressive subtype that remains difficult to treat [16, 17]. Due to the very aggressive nature of TNBC and the lack of well-established molecular therapeutic targets, patients with TNBC tend to have a relatively poorer outcome compared to patients with other subtypes [18, 19]. In breast cancer, and especially in TNBC, dissemination and metastatic growth of tumors at distant sites is the major cause of patient mortality [20]. Despite chemotherapy, fewer than 30% of women diagnosed with metastatic TNBC will survive beyond 3 years, and, unfortunately, almost all women with metastatic TNBC will ultimately succumb to their metastatic disease [21–23]. Although newer therapies and combinations of therapies for TNBC are under active investigation and hold future promise, including the use of poly (ADP-ribose) polymerase (PARP) inhibitors for TNBC patients with homologous recombination DNA repair-deficient cancers associated with *BRCA1* mutations, the use of immune checkpoint inhibitors, approaches that target other signaling pathways, or combination therapies, responses are still only observed in a small fraction of patients with advanced TNBC [24–30].

Factors that drive tumor metastasis have been a subject of intense scrutiny and research. As circulating tumor cells (CTCs) are considered contributory precursors that seed metastases in many cancers, including breast cancer, studying the biology of CTCs has provided vital clues regarding cancer metastasis [31]. Multiple mouse models may be used to study breast cancer biology, including syngeneic models (immunocompetent models generated from murine breast cancer cell lines, such as 4T1 cells), environmentally induced tumor models, transgenic models (models expressing mouse oncogenes, such as the polyomavirus middle T antigen controlled by the mouse mammary tumor virus long terminal repeat promoter, MMTV-PyMT model), genetically engineered mouse models (GEMMs), cell line-derived xenografts, and patient-derived xenografts [32–39]. However, the use of in vivo models to study the shedding and biology of human CTCs requires either human breast cancer cell line-derived xenografts [40] or patient-derived xenografts (PDXs). Generation of PDX models involves the transplantation of primary human cancer cells or pieces of tumor tissue into immunocompromised mice. Although most PDX models are generated in mice lacking a functional human immune system, they are still considered to be highly clinically relevant [41, 42], particularly when implanted orthotopically (e.g., human breast tumor tissue from the operating room implanted into the mammary fat pads of mice). Orthotopically implanted PDX models, called patient-derived orthotopic xenograft (PDOX) models, have been shown to recapitulate critical histological, genomic, transcriptomic, and proteomic features of the patients’ tumors from which they were derived [43–45]; they are also better models of human metastatic disease [46] and of response to anti-cancer therapies [41, 45, 47] and, in the case of TNBC PDOX models, represent more aggressive phenotypes [48]. Currently, there is renewed interest in utilizing PDX models as evident in US National Cancer Institute’s (NCI) plan to replace their NCI-60 cell line resource with PDX samples [49]. However, the background strain of mouse used for PDX studies is of key importance. NOD scid gamma (NSG) mice have been shown to be best at recapitulating the entire metastatic process in breast cancer from implantation in the mammary fat pad to distant metastatic spread, including development of metastases in cell line models that had not been previously associated with metastatic spread [50]. So while there has been an increasing interest in using PDOX models of breast cancer to study human CTCs captured from mouse blood and/or human disseminated tumor cells (DTCs) from mouse bone marrow [51–57], the studies from these other groups were performed in non-NSG mouse models. We had previously described the isolation of CTCs from two NSG mice from a single TNBC PDOX tumor [58]. In this study, we use NSG mice to examine the distribution of distant metastatic spread in 57 mice from seven PDOX models of TNBC and to analyze CTC shedding in 37 of these mice.

Multiple technologies have been developed to enable CTC isolation from the peripheral blood of human patients with solid tumors. Approaches to capture and/or identify these rare cells rely on positive or negative cell selection, density gradient centrifugation, microfiltration, microfluidic or electrophoretic separation, direct imaging, or functional assays [59–61]. Here, we isolate human CTCs from mouse blood in PDOX models using the Vortex platform, a technology originally developed to allow fast and label-free isolation of human CTCs over a broad range of blood volumes (200 μL to 16 mL). CTCs are enriched from whole blood based on size and deformability, using inertial microfluidics combined with microscale vortices [62]. This technique had been successfully used to isolate human CTCs from blood samples from patients with metastatic breast, lung, colorectal, and prostate cancer [63–67]. In the present study, isolation of human CTCs from mouse blood was first optimized using a human breast cancer cell line-derived xenograft model of TNBC, generated from MDA-MB-231 cells, that we had previously shown to shed CTCs and metastasize to the lung [40]. Based on these experiments, a set of seven PDOX models of TNBC, including four that had previously undergone global genomic and transcriptomic analyses [44], were used for replicate isolation and characterization of human CTCs and for identification of sites of distant metastases.

## Methods

### Vortex microfluidic chip design and operation

Vortex chips are 70-μm-deep microfluidic chips comprising a parallelized array of 16 straight channels, 40 μm wide, with each channel leading to a series of 12 (Vortex HT chips) or 9 (Vortex VTX-1 chips) rectangular trapping reservoirs (480 μm × 720 μm). Vortex HT PDMS chips were fabricated following conventional poly (dimethylsiloxane) (PDMS) fabrication processes with a 1:10 PDMS mix [68]. Vortex VTX-1 chips were fabricated with poly (methyl methacrylate) (PMMA) (Vortex Biosciences) using a standard lithography process [58]. The blood samples were injected through the chip as previously described [58]. Briefly, two syringe pumps (Harvard Apparatus) were connected to the chip using a plastic manifold, connectors (Upchurch), and tubing (Tefzel® (ETFE) Tubing Natural 1/16″ OD × .040″ ID from IDEX). One syringe was used for the blood sample and one syringe for the phosphate-buffered saline (PBS) wash buffer. After a priming step to fill the microfluidic path, the diluted blood sample was injected at 8 mL/min to enable the CTC enrichment. A washing step with PBS was then injected at a similar flow rate to remove contaminating blood cells. Stopping the flow finally released the CTCs from the vortices to their collection downstream in a well plate.

### Cancer cell line culture and harvesting

Cell lines used for characterization were grown aseptically to 30–60% confluence at 37 °C in a humidified atmosphere of 5% CO_2_. MDA-MB-231 (triple-negative breast carcinoma, ATCC® HTB-26™) cells were grown in DMEM medium supplemented with 10% inactivated FBS and 1% penicillin/streptomycin. Adherent cells were dissociated with TrypLE express cell dissociation reagent (Gibco) and resuspended in complete media. For experiments related to Fig. 2, the cell line used was MDA-MB-231 cells that stably expressed firefly luciferase-enhanced green fluorescent protein (FLuc-eGFP), a generous gift from the Paulmurugan lab at Stanford University [69].

### Cell immunostaining and enumeration

After Vortex processing of mice blood, cells were collected in a cell culture-treated 96-well plate (Nunc), fixed for 10 min with an equal volume of 4% paraformaldehyde (PFA; Electron Microscopy Sciences) for a final concentration of 2% PFA, permeabilized for 7 min with 0.4% Triton X-100 (Sigma Aldrich) volume/volume for a final concentration of 0.2%, blocked for 30 min with 10% goat serum (Invitrogen), and then stained for 1 h at room temperature (RT). The immunostains were 4,6-diamidino-2-phenylindole (DAPI) (Life Technologies), a rat anti-mouse CD45-PE antibody (CD45-PE, clone 30-F11, BD Pharmingen), and a cocktail of primary antibodies labeled with fluorescein isothiocyanate (FITC) to identify cytokeratin (CK)-positive cells (human anti-cytokeratin clone CK3-6H5, Miltenyi Biotec, and human anti-cytokeratin clone CAM5.2, BD Biosciences) [58]. For patient-derived orthotopic xenograft (PDOX) samples and MDA-MB-231 tumor xenograft samples, an anti-CK-AlexaFluor (AF) 488 antibody (human anti-pan cytokeratin clone AE1/AE3, eBioscience) and an anti-vimentin-AF647 antibody (clone V9, Abcam, reactive to human vimentin; non-reactive to mouse vimentin) were used in addition to the previously listed antibodies (Additional file 1: Figure S1). After staining, the cells were imaged using an Axio Observer Z1 microscope (Zeiss) and manually enumerated as described previously [58, 68]. Stitched images of stained cells were acquired, and cells enumerated by two different persons following a classification criterion developed at Vortex [58, 66, 68]. The cells were categorized into three large groups, namely CTCs (CK+/CD45−/DAPI+, VIM+/CD45−/DAPI+, and CK+/VIM+/CD45−/DAPI+), WBCs (CK−/CD45+/DAPI+, VIM−/CD45+/DAPI+, and VIM+/CD45+/DAPI+), or debris.

### CTC isolation from mouse blood

#### Cell spiking in healthy mouse blood

Two hundred to 500 MDA-MB-231 cells prepared as described above were spiked into 500 μL of blood isolated from healthy BALB/c mice via cardiac puncture and collected into EDTA-coated microtainer tubes. The spiked blood was diluted 10×, 20×, or 40× in filtered PBS and processed using the Vortex VTX-1 plastic chip. Once captured, the cells were released into a well plate, stained and enumerated. Capture performance can be described by cancer cell recovery and capture contamination using the following equations:

$$ \mathrm{Cell}\ \mathrm{recovery}\ \left(\%\right)=\frac{\#\mathrm{of}\ \mathrm{target}\ \mathrm{cells}\ \mathrm{captured}}{\#\mathrm{of}\ \mathrm{target}\ \mathrm{cells}\ \mathrm{spiked}\ \mathrm{into}\ \mathrm{the}\ \mathrm{sample}} $$

$$ \mathrm{C}a\mathrm{pture}\ \mathrm{contamination}\ \left(\mathrm{WBCs}/\mathrm{mL}\right)=\frac{\#\mathrm{of}\ \mathrm{WBCs}\ \mathrm{captured}}{\mathrm{volume}\ \mathrm{of}\ \mathrm{blood}\ \mathrm{processed}} $$

#### PDOX samples

At time of euthanasia and necropsy, blood (~ 750 μL) was collected via cardiac puncture from PDOX-bearing mice and diluted 40× with filtered PBS. The diluted samples were processed through Vortex VTX-1 plastic chips, and the isolated CTCs were immunostained and enumerated as described above.

### Animal studies

All studies were approved by the Stanford University Research Compliance Office’s Human Subjects Research and IRB Panel and Stanford’s Administrative Panel on Laboratory Animal Care (APLAC). All methods were performed in accordance with the relevant guidelines and regulations. All seven TNBC PDOX tumor models were generated from fresh-frozen live tumor tissue fragments from our bank of previously established patient-derived orthotopoic xenograft tumor models, including four models of different TNBC subtypes that were previously characterized using global genomic and transcriptomic analyses [44]. Tumor samples were collected from patients in accordance with the relevant IRB guidelines. Briefly, frozen fragments of tumor were thawed and washed once with RPMI-1640 media and transported under aseptic conditions to the Stanford animal facility. One to 2 mm fragments of individual PDOX tumors were then sterilely and orthotopically transplanted into the fourth mammary fat pads of at least 5 female NOD *scid* gamma (NSG) mice (NOD.Cg-*Prkdc*^*scid*^ *Il2rg*^*tm1Wjl*^/SzJ (Jackson Laboratory West, Sacramento, CA, USA). These mice have deficiencies in innate immunity, and due to the severe combined immune deficiency mutation (*scid*) and an IL2 receptor gamma chain deficiency that disables cytokine signaling, they lack mature T cells, B cells, and functional NK cells and are deficient in cytokine signaling. In contrast to the more leaky NOD *scid* mice, NSG mice have a longer lifespan with more resistance to lymphoma.

Animals were anesthetized using 1 to 3% isoflurane, hair at the implantation site was removed, and skin was sterilized with povidone-iodine and alcohol. A small skin incision was made, and the fourth mammary fat pad was identified. The fat pad was gently held with forceps while a small nick was made to create a pocket where tumor fragments were then placed. The skin incision was closed using interrupted monofilament sutures. Mice were maintained in pathogen-free animal housing. For MDA-MB-231 xenograft studies, a mixture of cell suspension and Matrigel (LDEV-free, Growth Factor Reduced, BD Biosciences) was injected into the fourth mammary fat pad. All the animals were monitored regularly, and tumor growth was measured at regular intervals. Tumor volume was calculated by the following formula: tumor volume = (*l* × *w*^2^)/2, where *l* was the longest diameter of the tumor and *w* was the shortest diameter of the tumor. Mean tumor volumes were calculated, and growth curves were established as a function of time. All animal care was performed in accordance with IACUC and Stanford University Administrative Panels on Laboratory Animal Care guidelines (APLAC Protocol #12809).

For testing the impact of route of blood collection on CTC numbers, the TNBC xenograft models used were generated from MDA-MB-231 human breast cancer cells that stably expressed firefly luciferase-enhanced green fluorescent protein (FLuc-eGFP), as mentioned above, with tumor cells and CTCs showing green fluorescence. Briefly, 8 × 10^6^ MDA-MB-231-FLuc-eGFP cells were injected orthotopically into the fourth mammary fat pads of NSG mice (*n* = 35). Tumor volumes were measured three times per week for all the animals. Starting from week 1 post injection, blood via cardiac puncture (500 μL) (*n* = 3 animals) or via lateral saphenous vein (100 μL) (*n* = 3 animals) was collected weekly, diluted to 40×, and processed on the Vortex platform to enrich for CTCs. After staining, CTCs were enumerated. For testing the retroorbital route of blood collection, blood from non-tumor-bearing NSG control mice was collected, processed on the Vortex platform, and stained for DAPI, cytokeratin, and CD45.

### H&E analyses for metastases

Upon termination of the experiment, animals bearing PDOX tumors were humanely euthanized via CO_2_ asphyxiation. Blood, tumor tissue, and organs were collected immediately following euthanasia. Lungs from individual animals were inflated with 10% neutral buffered formalin (NBF) prior to submersion and storage in 10% NBF. All organs were immersion-fixed in 10% NBF for at least 48 h prior to transferring to 70% ethanol. Fixed sections of the lung, liver, and brain were routinely processed and embedded in paraffin, then serially sectioned at 5 μm and stained with hematoxylin and eosin (H&E; HISTO-TEC laboratories, Hayward, CA). Five serial H&E sections of each individual organ were then screened for tumor metastases at × 4 and × 40 magnification (Zeiss Axioskop 2 Plus) by board-certified veterinary pathologists (KMC and JGMV). Photomicrographs of metastases were captured and visualized using a Nikon DS-Ri1 camera and NIS-Elements Imaging Software (2011), respectively.

## Results

### Optimization of CTC isolation from mouse blood samples with spiking experiments

#### Effect of blood dilution

**Fig. 1:**
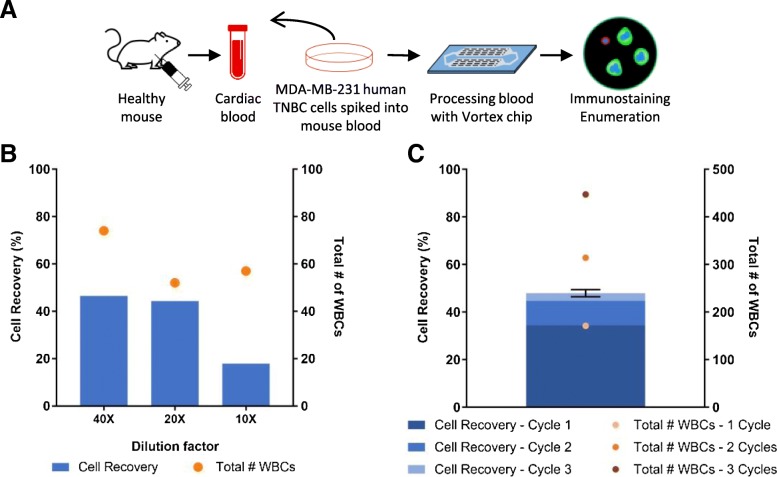
Workflow optimization for the isolation of human cancer cells spiked into healthy mouse blood using Vortex technology. **a** Workflow schematic, from blood collection to cell spiking into blood, cell isolation, and downstream immunostaining. **b** Effect of blood dilution on the performance of cancer cell isolation (*n* = 1). **c** Effect of blood efflux recycling on cancer cell recovery and WBC contamination (blood dilution factor of 40×) (*n* = 3)

Mouse blood is less viscous than human blood [70] but of significantly smaller total volume, usually less than 2 mL per animal. Small volumes of collected blood pose a challenge for processing through fluidic tubing and microfluidic components because of the risk of loss of rare target cells in dead volume. To overcome this issue, diluting the blood sample becomes essential. In a previous study, different dilutions of human blood were tested on the Vortex technology, and the results demonstrated that a 10× dilution was optimal for cancer cell recovery and throughput [62]. To establish a dilution strategy for mouse blood, 200 MDA-MB-231 human breast cancer cells were spiked into 500 μL of healthy mouse blood, diluted with PBS (40×, 20×, and 10×) and processed on the Vortex platform (Fig. 1a). As illustrated in Fig. 1b, a similar cancer cell recovery was obtained for 40× and 20× dilutions (46.55% and 44.34% recovery) and 74 and 52 WBCs were also captured. With the 10× dilution, the recovery was poor (17.89%). Based on these results, a 40× dilution of mouse blood was used for all ensuing experiments.

#### Effect of recycling the blood efflux

To optimize CTC recovery from a small blood volume, we devised a strategy of recycling, wherein after processing once (first cycle), the efflux is collected and reinjected into the microfluidic chip for a second and third time (second and third cycles), to capture additional cancer cells that might have escaped capture in the first round of processing (Fig. 1c). For testing the efficiency of recycling, 500 MDA-MB-231 cells were spiked into 500 μL of mouse blood and processed through the microfluidic chip for up to three cycles. When processed in the High Purity Mode (one cycle), 34.38% of cells were collected on average. Cycling a sample twice captured 44.73% of the cancer cells. When the same sample was processed in the High Recovery Mode (three cycles), 47.96% of the cells were recovered. However, recycling also increased the number of co-isolated WBCs (447 WBCs with three cycles combined, compared to 171 with one cycle). For the following experiments, two cycles were selected as optimal.

### Effect of blood collection site on CTC capture

**Fig. 2:**
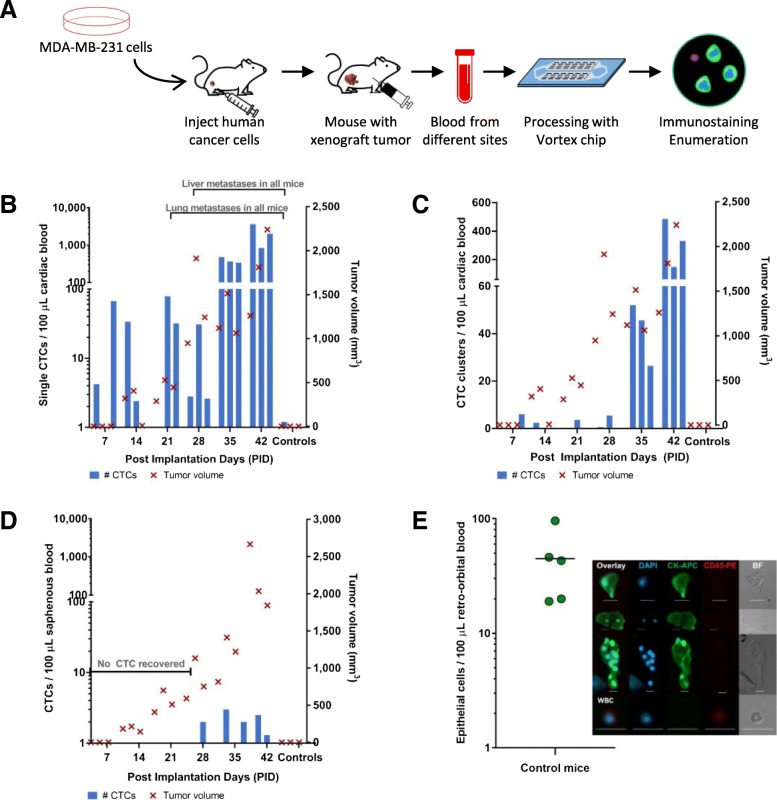
Blood collection site for CTC isolation from mouse blood using a MDA-MB-231 TNBC xenograft model. **a** Workflow schematic. **b**, **c** Tumor growth and numbers of CTCs, including counts of single CTCs in panel **b** and CTC clusters in panel **c**; blood was obtained by cardiac puncture in sets of three mice weekly after implantation. **d** Saphenous vein collection provided less CTCs despite relatively similar tumor growth over time. **e** CK+ epithelial cells were identified in retroorbital blood collected from control mice without tumors

Blood samples from mice bearing MDA-MB-231 tumor xenografts were collected at different time intervals from the saphenous vein or by cardiac puncture as tumor size increased (Fig. 2). CTCs were detected in blood collected via cardiac puncture as early as day 7. CTC counts ranged from 0.4 to 3649 CTCs/100 μL and increased over time, correlating with tumor burden (Fig. 2b). CTC clusters were also captured from day 7 (6 clusters/100 μL; frequency 1/3), with number and frequency increasing over time up to 147–485 clusters/100 μL by day 42 (Fig. 2c). In contrast, no CTCs were recovered from blood obtained from the lateral saphenous vein until day 28 post-implantation, and their number remained low (mean 2.15 ± 0.65 CTCs/100 μL) (Fig. 2d). Blood collected from the retroorbital sinus from healthy control animals without tumors had high numbers of contaminating epithelial (CK+/DAPI+/CD45−) cells making this route of blood collection unsuitable for CTC studies (Fig. 2e).

### Characterization of tumor growth, metastases, and CTCs in PDOX models of triple-negative breast cancer

**Fig. 3:**
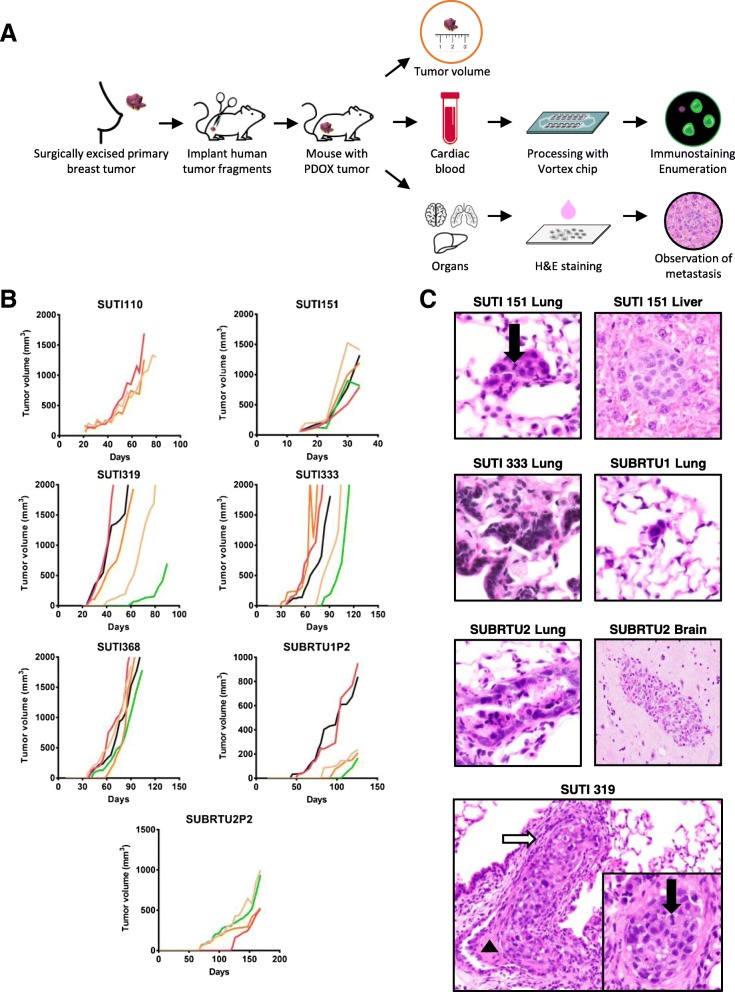
Growth rate and metastases in PDOX models of TNBC. **a** For individual animals, tumor fragments from previously derived and passaged PDOX tumors were implanted orthotopically in the fourth mammary fat pad and tumor volume was measured over time; at the end of the experiment, cardiac blood was collected for CTC isolation, and multiple organs were analyzed for metastases. **b** Representative growth curves for seven different PDOX tumor models grown in NSG mice. **c** Metastases were identified in the lung, liver, and rarely, brain of mice bearing the human TNBC tumors (H&E, × 20 and × 40 magnifications). Pulmonary metastases were found within alveolar septal capillaries or medium- to small-caliber vessels, where they were associated with peri-tumoral fibrin (black triangle) and/or desmoplasia (white arrow). Hepatic metastases were randomly distributed within hepatic sinusoids. Mitotic figures (black arrows) were frequently identified within metastatic foci

After implantation of fragments of TNBC PDOX tumor tissue into NSG mice, individual tumor volume was calculated and plotted as a function of time; after sacrifice, organs were examined and, in a subset of mice, blood was processed for CTCs (Fig. 3a). Different PDOX tumor models grew at different rates, and there were differences in tumor growth rates in individual mice bearing the same PDOX tumors (Fig. 3b).

To test for metastatic spread in our TNBC PDOX models, the lung, liver, and brain were screened for metastases by serial section and hematoxylin and eosin (H&E) staining (Fig. 3c). Metastases were detected in the lung (20/57, 35%), liver (7/57, 12%), and brain (1/57, 2%), with different TNBC PDOX models displaying varying sites and frequencies of metastases (Table 1). For example, PDOX tumor model SUTI151 displayed a high frequency of metastases with two thirds of the animals bearing this tumor showing metastases in at least one organ. Interestingly, this TNBC PDOX model was the most aggressive, derived from a patient’s rapidly metastasizing breast cancer that was refractory to all standard therapies; the SUTI151 model also exhibited the highest number of CTCs per tumor-bearing mouse compared to other PDOX models in this study. We detected a brain metastasis in only one PDOX tumor model, SUBRTU2 (Fig. 3c and Table 1). Notably, one PDOX model in our cohort, SUTI368, showed no metastases at necropsy.

**Table 1:**
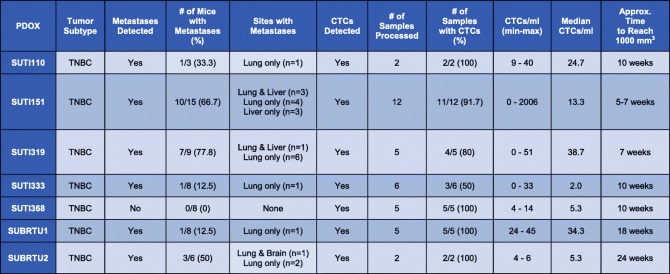
Tabulated results of metastases detected in 57 mice bearing seven different TNBC PDOX tumor models. Thirty-seven blood samples obtained by cardiac puncture from these seven TNBC tumor models were processed for CTC isolation

Histologically, metastases were noted in association with vascular structures of the lung and liver (Fig. 3c). Within the lung, neoplastic thromboemboli were found in medium- to small-caliber pulmonary vessels (Fig. 3c, × 20 magnification), as well as within capillaries of the alveolar septa (Fig. 3c, × 40 magnification). Within medium- to small-caliber vessels, neoplastic thromboemboli partially to completely occluded vascular lumina and were segmentally adherent to the vascular endothelium. Occasionally, there was disruption of the vascular wall, and associated luminal and extraluminal fibrin deposition (Fig. 3c, black triangle). Within the lungs, neoplastic aggregates were occasionally associated with concentric bands of fibrous connective tissue (desmoplasia, Fig. 3c, white arrow). Within the liver, discrete neoplastic aggregates were randomly distributed within the sinusoids of the hepatic parenchyma. Concomitant desmoplasia was not noted within any of the examined sections of the liver. Neoplastic cells were polygonal with distinct cell borders and a low to moderate amount of eosinophilic cytoplasm. Nuclei were round to ovoid with finely stippled chromatin and up to one discrete nucleolus. Variation in cell size (anisocytosis) and nuclear size (anisokaryosis) were moderate, and mitotic figures were frequent (Fig. 3c, black arrows). Occasional bizarre mitotic figures were noted. Rarely, individual cell necrosis was characterized by contracted hypereosinophilic cytoplasm and karyorrhectic to pyknotic nuclei.

#### CTCs in TNBC PDOX models

**Fig. 4:**
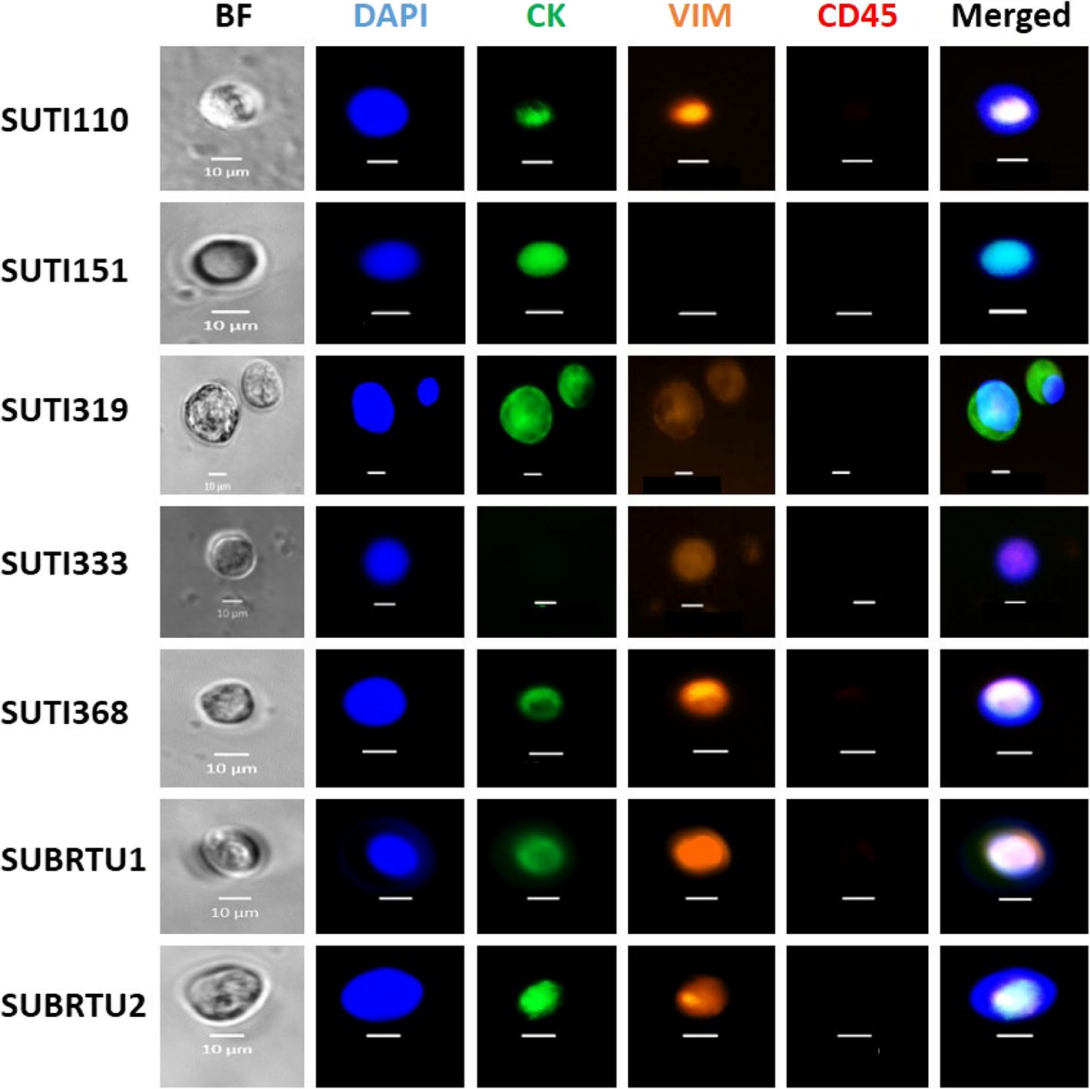
Representative fluorescent images of CTCs isolated from different PDOX tumor-bearing mice. Live CTCs were isolated from individual PDOX tumor-bearing animals by label-free Vortex technology. Captured cells were then fixed, permeabilized, and stained for human cytokeratin (CK), human vimentin (VIM), and mouse CD45. Nuclei were highlighted with DAPI. CTCs were identified as DAPI-positive cells that were positive for cytokeratin and/or vimentin and negative for CD45 (to exclude mouse hematopoietic cells)

To test for the presence of CTCs in our seven different TNBC PDOX models, we processed whole blood isolated via cardiac puncture from 37 of the PDOX tumor-bearing mice using the Vortex CTC isolation platform (Fig. 3a). Staining and imaging of captured cells revealed the presence of CTCs in all seven PDOX models tested, even in the SUTI368 model that showed no distant metastases (Fig. 4, Table 1). The total number of CTCs varied between different PDOX tumor models and between individual mice bearing the same PDOX tumor model (Table 1).

#### Isolation of CTC clusters

**Fig. 5:**
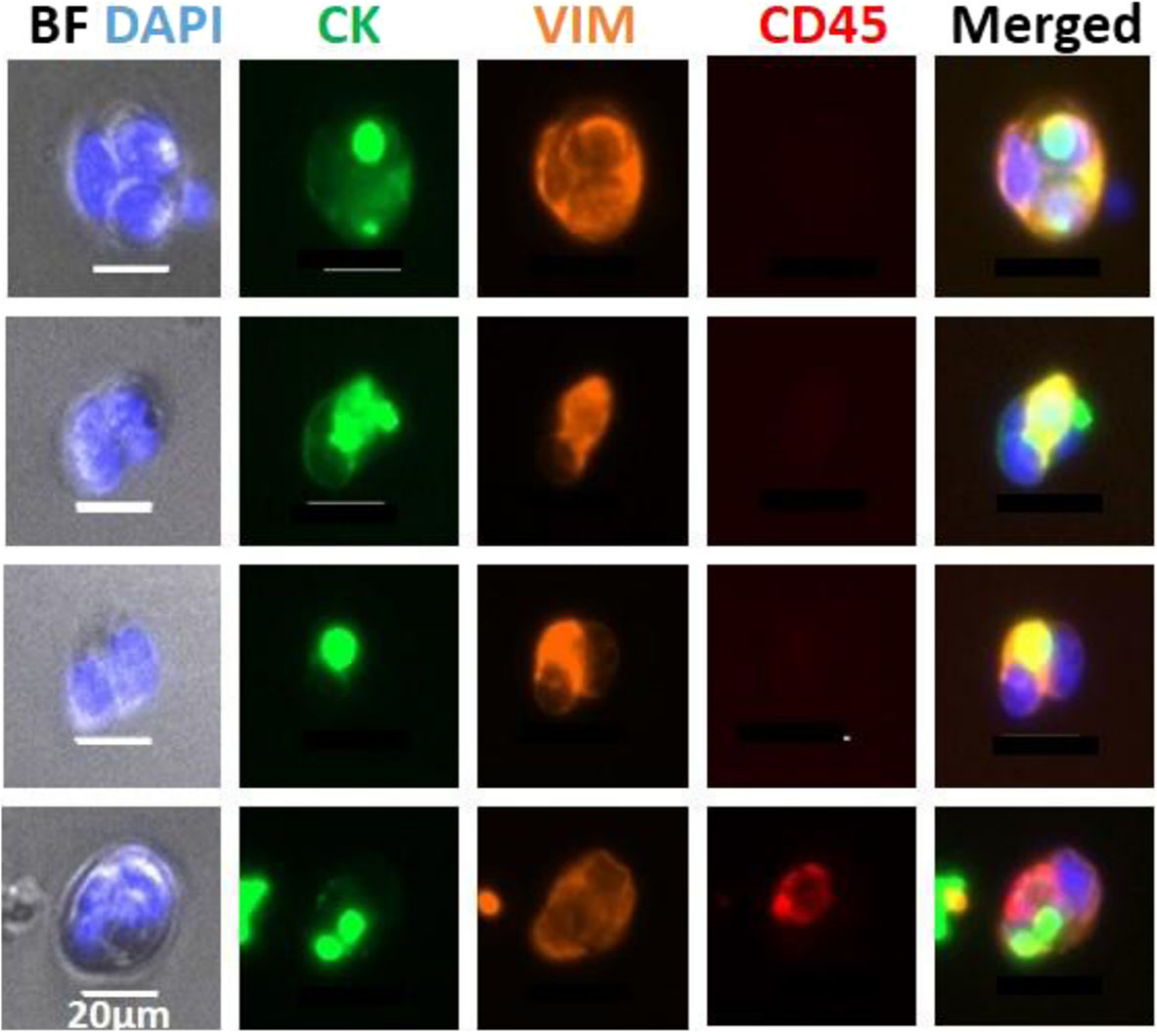
CTC clusters from mice bearing MDA-MB-231 xenograft tumors. CTC clusters were isolated using the Vortex platform and stained with DAPI, human cytokeratin (CK), human vimentin (VIM), and mouse CD45. CTCs within clusters were identified as cells positive for DAPI, cytokeratin, and/or vimentin and negative for CD45. Note the heterogeneous composition of cells within some clusters

We found that both our MDA-MB-231 cell line-derived and PDOX models revealed the presence of CTC clusters (Fig. 2c and Fig. 5), and only one of our PDOX models, SUTI151, revealed the presence of CTC clusters. Importantly, some of the captured CTC clusters from this model displayed a clear heterogeneity in cytokeratin and vimentin staining among the individual cells present within the cluster.

#### CTCs from TNBC PDOX models showed markers of epithelial, mesenchymal, and mixed phenotypes

**Fig. 6:**
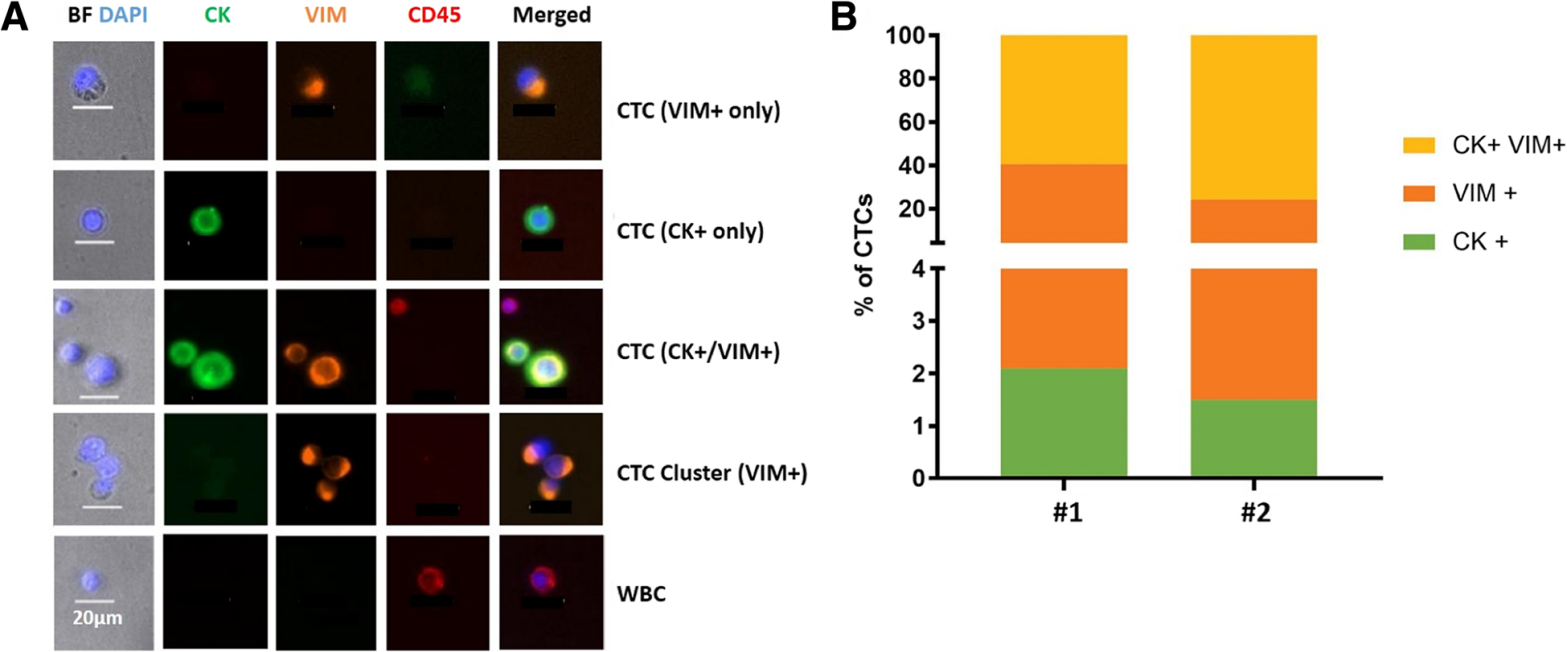
Epithelial, mesenchymal, and mixed phenotypes of CTCs isolated from a metastatic TNBC PDOX model. **a** Fluorescent images of CTCs isolated from a mouse bearing PDOX tumor SUTI151 captured using the Vortex platform and probed for the epithelial marker (cytokeratin, CK) and the mesenchymal marker (vimentin, VIM). Nuclei were highlighted with DAPI. CTCs were identified as DAPI positive, positive for CK and/or VIM, and negative for CD45. **b** Graph representing the percentage of CTCs staining positive for CK and/or VIM from two individual mouse models (#1, #2) bearing the same PDOX SUTI151 tumor, with the majority of CTCs showing a mixed epithelial and mesenchymal phenotype

CTCs detected in our different PDOX tumor models of TNBC expressed basic epithelial (CK+) and/or mesenchymal (VIM+) markers, suggesting that individual CTCs may be in the process of undergoing epithelial-mesenchymal transition (EMT) (Figs. 4 and 6). Quantifying CTCs isolated from the TNBC PDOX model SUTI151 demonstrated that while some CTCs showed an epithelial phenotype (cytokeratin-positive, vimentin-negative) or a mesenchymal phenotype (cytokeratin-negative, vimentin-positive), the great majority of CTCs from this model expressed a mixed phenotype, positive for both cytokeratin and vimentin (Fig. 6a). Grouping the CTCs into distinct subsets based on the expression of these markers from two individual mice bearing SUTI151 tumors (as mentioned above, this model was derived from a particularly aggressive TNBC that metastasized rapidly) showed that the percentage of CTCs in each subset between the two animals were fairly similar (Fig. 6b). While such results highlight the utility of PDOX models for further investigations of EMT in CTCs, a strong correlation of EMT and the ability of CTCs to generate metastases would require further thorough investigation involving multiple PDOX models that display EMT changes. Here, it was only seen in one of our seven TNBC PDOX models.

## Discussion

Circulating tumor cells, detected most often in metastatic cancer [71], are a key player in the metastatic cascade [31]. We have recently reported on label-free isolation and analysis of CTCs from two PDOX tumor-bearing mice as part of an evaluation of Vortex’ fully automated VTX-1 Liquid Biopsy System [58]. Here, we investigated CTC shedding and metastases in TNBC PDOX models, examining 57 mice from a cohort of seven TNBC PDOX models for distant metastases in serially sectioned lung, liver, and brain, and using a marker-agnostic CTC isolation platform to isolate and analyze CTCs from 37 of these mice.

As far as we are aware, seven previous studies have used breast cancer PDOX models to capture and analyze CTCs from mouse blood or DTCs from mouse bone marrow. One study [54] used a previous *TP53* wildtype PDOX model of non-basal TNBC to generate cell lines that were transduced with lentivirus encoding CBR-luc and mCherry (FUW-CBR-luc-mCherry) and then infected with control retroviruses or retroviruses encoding p53-specific short hairpin RNAs (shRNAs) to knock down p53. They then implanted these wildtype and p53-deficient transduced cells into cleared mammary fat pads of NOD/SCID mice whose stroma was humanized using immortalized and irradiated human mammary stromal fibroblasts derived from a patient undergoing a reduction mammoplasty. Tumor growth; metastases to the lung, bone, liver, brain, and axillary lymph node; and CTC shedding (quantified using flow cytometry) were compared between wildtype and p53-deficient tumors. Although their PDOX models were generated very differently than ours, similar to our findings, metastatic tumor was identified at different sites in differing fractions of mice. Moreover, although CTC shedding increased with time, a finding more pronounced in mice whose tumors were p53-deficient, with numbers of CTCs in the less than 15 range at 9 weeks to the less than 220 range at 18 weeks, CTC shedding appeared more related to total primary and metastatic tumor burden. A different study [55] used PDOX models derived from five tumors of different breast cancer molecular subtypes that were implanted in humanized mammary fat pads (previously cleared and injected with GFP-labeled immortalized human fibroblasts) of NOD/SCID mice to investigate DTCs and distant metastases. Here, mouse bone marrow was analyzed by performing qRT-PCR for human transcripts as well as microarray analysis of gene expression in primary tumors, the bone marrow, and a metastatic lesion in one mouse. Only two PDOX tumor models developed metastases, both of which had human DTCs and which had variable sites of metastases. Global gene expression between diverse sources in the one mouse tested, a TNBC PDOX model, was also variable, but seemed to show patterns of human gene expression “reminiscent of a ‘mesenchymal-like’ phenotype in multiple animals across multiple passages,” with the authors suggesting that DTCs from the bone marrow may have undergone a phenotypic transition that enabled migration to and survival in the bone marrow, similar to many of the CTCs we observed in our current study. Another study [52] measured CTCs from blood and DTCs from the bone marrow, detected using anti-human pan-cytokeratin immunohistochemistry (IHC), in 18 different breast cancer PDOX models, 13 of which were TNBC PDOX models, generated from human breast tumors implanted into the cleared mammary fat pads of SCID/beige mice. Again, differing fractions of individual mice in each model had detectable CTCs (overall, present in 83% of PDOX models) and/or DTCs (overall, present in 63% of PDOX models) and lung metastases (overall, present in 50% of PDOX models). Although no specific mesenchymal-like IHC marker was used, there was still a strong association between CTC and DTC detection. Importantly, all mice with lung metastases had detectable CTCs but not all mice with detectable CTCs had lung metastases, similar to our findings. A strong association between CTC clusters and the presence of lung metastases was also found. In a fourth study [51], single-cell suspensions of seven patient-derived breast tumors were orthotopically injected into SCID mice; two PDOX tumors developed CTCs as detected by an EpCAM-based platform, and no metastases were observed in any of the seven mice. An important study by the Werb group [53] using three PDOX models of TNBC in NOD/SCID mice showed that all models shed CTCs into blood, with variable CTC shedding (7/31 mice = 23% in one TNBC model had detectable cancer cells in their peripheral blood; 1/19 mice = 5% in another TNBC model had detectable cancer cells in their peripheral blood; and 3/22 mice = 14% in a third TNBC model had detectable cancer cells in their peripheral blood). All models developed metastases in different organs, but again, like our study, showed variability among individual mice within a TNBC model. Excitingly, single-cell gene expression signatures of cells from tissues with low and high metastatic burden and CTCs showed that a subpopulation of stem-like CTCs may potentially represent “metastatic seeder cells.” A recent study of CTCs in non-NSG mice [56] used the same PDOX models described in their previous work [45, 52] but with CTCs isolated using a different CTC isolation platform that improved on CTC number and allowed identification of the *PIK3CA* T1035A mutation in single cells from CTCs, primary tumors, and lung metastases in one of their PDOX models. Finally, an interesting new study that analyzed human RNA transcripts in the blood (positive for CTCs), bone marrow (positive for DTCs), and tumor in severe combined immunodeficient (SCID) xenograft mouse models found that CTCs from an ER-positive PDOX model of lobular carcinoma expressed a possibly uncoupled epithelial-mesenchymal plasticity and lower expression of stem cell-like markers than CTCs from a cell line-derived TNBC xenograft [57].

A challenge when studying in vivo CTC models has been identifying an appropriate route of blood collection for maximal CTC yield for downstream analyses. We addressed this question first by using a metastatic, human TNBC cell line, MDA-MB-231, and discovered that blood collected via cardiac puncture yielded higher numbers of CTCs and CTC clusters, with some clusters heterotypic and containing both CTCs and WBCs. The importance of blood collection route for CTC analyses in mouse xenograft models was nicely addressed using cell line-derived xenografts and an EpCAM-based CTC isolation platform [51], concluding that cardiac puncture was the best route for obtaining CTCs compared to tail vein, retroorbital, and jugular vein aspiration. Similar to our findings, blood samples from the retroorbital venous plexus were uniformly contaminated by CTC-like normal murine epithelial cells in control mice without tumors. They also found that jugular vein aspiration yielded CTCs in only 15% of samples and also showed normal murine epithelial cell contamination from jugular vein aspiration in control mice. To our knowledge, our results are the first to address blood collection route using a marker-agnostic CTC isolation platform. While the cardiac route of blood collection is superior in its CTC yield, a major limitation of this route is that, as used here, it is a terminal method of blood collection, thereby limiting its application for serial CTC monitoring.

Analyzing CTCs and distant metastases in different breast cancer PDOX models also depends on the genetic background of the mouse model selected. A recent study highlighted challenges and limitations of metastatic TNBC PDOX models using SCID mice [72]. The authors discussed several challenges that included low rate of metastases, long periods for metastases in the lungs to be detected, and development of thymic lymphomas of mouse origin, using their SCID mouse models. SCID mice may also develop spontaneous non-thymic tumors, including mammary adenocarcinomas [73]. Pertinently, while younger NSG models do not develop spontaneous tumors, a recent study of aging female NSG mice showed that tumors may indeed develop in mice of median age 52 weeks, when age was available, including 31% developing spontaneous mammary gland neoplasms [74], a recent finding for which the PDOX community should be made aware.

In another study using NCr nude and SCID mice, only 2/7 PDOX models yielded CTCs and none had detectable distant metastases [51]. Addressing the issue of mouse model background, the Lippman group showed that NSG mice are superior for generating distant metastases in cell line- and patient-derived orthotopic xenograft models [50]. Using NSG mice for our study, most PDOX tumors grew aggressively and produced large tumors within 3 months of implantation, with detectable metastatic lesions in 6/7 of our TNBC PDOX tumor models.

In our study, the percentage of PDOX tumor-bearing mice with metastases ranged from 78% for SUTI319 to 0 for SUTI368. It is interesting to note that although CTCs were detected in 5/5 of SUTI368 tumor-bearing mice, no distant metastases were detected in eight SUTI368 PDOX mice. While serial sectioning of organs was sufficient to detect metastases in the majority of our PDOX models, we may have missed micrometastases in un-sectioned portions of an organ that perhaps a more sensitive PCR-based approach, based on detecting human DNA in mouse whole organs, could have potentially detected, including PDOX model SUTI368 [75]. However, a more likely explanation for the presence of CTCs in 5/5 mice and lack of metastases in 0/8 mice from this model is that *metastasis is an inefficient process* [76]. In a classic murine melanoma model with intravital video-microscopy, metastatic inefficiency was found to be mainly due to (1) lack of initiation of growth (i.e., dormancy) of solitary cancer cells in a distant organ and (2) lack of growth of initial micrometastases into macroscopic tumors and vulnerability to early destruction [77]. This has also been confirmed in multiple other tumor models, including breast cancer [78], and additional mechanisms have been proposed as to why only a subset of circulating tumor cells may be associated with the development of metastases [79–81].

While the SUTI368 model is capable of generating CTCs, the tumor cells in circulation may not be capable of establishing metastatic growth because they may lack the ability to initiate the critical tumor-host interactions or may not possess intrinsic biomechanical or other molecular properties necessary for establishing metastases [82–85]. Moreover, it is interesting to note that even when all the individual animals were implanted with tumor tissue fragments from the same PDOX tumor, there were differences in tumor growth rates between the individual tumor-bearing animals as well as differences in the development of and site of metastases within the same PDOX tumor model. Heterogeneity in the amount of tumor tissue and associated stromal tissue in each fragment implanted, differences in the number of viable tumor cells, and the molecular heterogeneity of tumor cells in individual tumor fragments could trigger such different growth rates in vivo. To overcome such discrepancies, we are also using single-cell suspensions of tumor tissue for generating and passaging more uniform PDOX tumors (data not shown).

CTCs can exist as single cells or clusters, and CTC clusters are predicted to have a greater potential to establish metastases than individual CTCs [86–90]. In mouse models of breast cancer, CTC clusters appear to be derived from oligoclonal groupings of primary tumor cells and have been shown to be a highly metastasis-competent subset of CTCs, compared to single circulating breast cancer cells [86, 87]. CTC clusters also may provide additional prognostic value in human breast cancer [91–93], and in patients with breast, prostate, and small cell lung cancer, the presence of single or increasing numbers of CTC clusters in sampled blood is found to correlate significantly with reduced progression-free survival rates [86, 91–96]. As a result, there has been an interest in isolating and understanding CTC clusters in different cancers and, in addition to filter and other technologies that identify clusters, some CTC isolation technologies have been specifically designed for isolating CTC clusters [97–99] or exploring their behavior [100]. In our studies, using the Vortex CTC isolation platform, we demonstrate rapid and efficient isolation of CTCs and CTC clusters from both cell line-derived xenograft and PDOX models of breast cancer. As this platform is marker-agnostic, we were able to capture heterogeneous clusters of CTCs that included individual cells that varied in the expression of CTC markers tested, demonstrating its utility for examining the biology and heterogeneity of CTC clusters in different cancer models.

Another notable finding in our current study is the difference in the heterogeneous distribution of cytokeratin (epithelial), vimentin (mesenchymal), and mixed cytokeratin and vimentin markers in CTCs and CTC clusters within and between the TNBC PDOX tumors. For example, while some CTCs from these PDOX tumor models showed high expression of the epithelial marker cytokeratin or the mesenchymal marker vimentin, many CTCs stained strongly for both cytokeratin and vimentin. The distribution of epithelial-like, mesenchymal-like, and mixed epithelial and mesenchymal CTC phenotypes represents an important aspect of CTC heterogeneity. CTC heterogeneity, including differences in gene expression of vimentin and five other EMT markers, was previously highlighted by our group using high-dimensional single-cell transcriptional profiling of human CTCs from breast cancer patients, where cell-to-cell heterogeneity of CTCs was noted even within the same blood draw [101]. This was also demonstrated through the work of other groups that has shown mixtures of epithelial- and mesenchymal-like CTCs in human blood samples of breast and other cancers [102–104]. It has been postulated that hybrid EMT phenotypes may promote the development of cancer metastases [105–107], although how the role of EMT plasticity and the different EMT states precisely contribute to the metastatic process still remains under active investigation [108–114]. Heterogeneity among CTCs and resulting metastases may also be attributed to (i) differences in methylation patterns of specific genes [115], (ii) selective and differential expression of particular genes in a subset of CTCs [101, 116–119], and/or (iii) expression of certain genes at different stages of the disease [120–123].

## Conclusion

Our data describes the heterogeneous distribution of lung, liver, and brain metastases in a group of seven TNBC PDOX models and confirms the shedding of CTCs captured by a marker-agnostic CTC isolation platform. It supports further use of PDOX models, generated in NSG mice, to study PDOX-derived CTCs, distant metastases, and for testing the impact and outcome of different anti-cancer agents on CTC shedding and metastasis in breast cancer.

## Additional file

Additional file 1: **Figure S1.** As a staining specificity control, human tumor cells from the MDA-MB-231 human TNBC cell line and mouse tumor cells from the 4T1 mouse TNBC cell line were both stained with the same set of antibodies used in the current study. The anti-pan cytokeratin antibody cocktail and the anti-vimentin antibody strongly stained human tumor cells; 4T1 mouse tumor cells showed minimal or absent staining with the same set of antibodies. (DOCX 234 kb)

## Data Availability

All data generated or analyzed during this study are included in this published article and can be shared by contacting the corresponding authors.
