# Supplementary material for: Investigating circulating tumor cells and distant metastases in patient-derived orthotopic xenograft models of triple-negative breast cancer
Source: Breast Cancer Res. 2019 Aug 28;21:98. doi: 10.1186/s13058-019-1182-4 (PMC6714238; doi:10.1186/s13058-019-1182-4)
Supplement: Supplementary file 1 — Figure S1. As a staining specificity control, human tumor cells from the MDA-MB-231 human TNBC cell line and mouse tumor cells from the 4T1 mouse TNBC cell line were both stained with the same set of antibodies used in the current study. The anti-pan cytokeratin antibody cocktail and the anti-vimentin antibody strongly stained human tumor cells; 4T1 mouse tumor cells showed minimal or absent staining with the same set of antibodies. (DOCX 234 kb) [file 13058_2019_1182_MOESM1_ESM.docx]

**Additional file 1:**

**Figure S1**. As a staining specificity control, human tumor cells from the MDA-MB-231 human TNBC cell line and mouse tumor cells from the 4T1 mouse TNBC cell line were both stained with the same set of antibodies used in the current study. The anti-pan cytokeratin antibody cocktail and the anti-vimentin antibody strongly stained human tumor cells; 4T1 mouse tumor cells showed minimal or absent staining with the same set of antibodies.
